# Middle-preserving pancreatectomy for multifocal intraductal papillary mucinous neoplasms of the pancreas: report of a case

**DOI:** 10.1007/s12328-014-0472-8

**Published:** 2014-03-19

**Authors:** Masaaki Nishi, Hideki Kawasaki, Masahiko Fujii, Miya Nagahashi, Masayoshi Obatake, Makoto Shirai, Koji Yamamoto, Masamitsu Harada

**Affiliations:** Department of General and Digestive Surgery, Ehime Prefectural Central Hospital, 83 Kasuga-cho, Matsuyama, Ehime 770-8503 Japan

**Keywords:** Middle-preserving pancreatectomy, Multifocal IPMNs

## Abstract

Multifocal or continuous pancreatic lesion is identified frequently but finding an appropriate surgical approach is quite challenging. Total pancreatectomy is a useful procedure. However, postoperative endocrine and exocrine disturbance is inevitable. Recently, the safety and feasibility of parenchyma preserving pancreatectomy, including middle-preserving pancreatectomy (MPP), have been reported. MPP is a combined procedure of pancreaticoduodenectomy and distal pancreatectomy, while preserving the body of the pancreas, for cases of multifocal pancreatic lesions. So far, there have only been a few reports that have described MPP. We report a case of MPP for multifocal intraductal papillary mucinous neoplasms of the pancreas, describe the surgical procedure, and discuss the feasibility of MPP as parenchyma-preserving pancreatectomy with reference to the literature.

## Introduction

Total pancreatectomy (TP) is a treatment option for multifocal or continuous lesions from head to tail of the pancreas. However, the oncological benefit of TP has not been established for pancreatic cancer. TP results in a complete loss of pancreatic function. Thus, postoperative endocrine and exocrine disturbance is inevitable. After TP, patients usually have severe diabetes, diarrhea, or malabsorption. Therefore, in choosing this treatment, the balance should be considered between oncologic outcome, life expectancy, postoperative complication, and quality of life.

Recently, there have been an increasing number of pancreatic surgeries. The safety and feasibility of parenchyma-preserving pancreatectomy (i.e., middle-preserving pancreatectomy [MPP], middle pancreatectomy, enucleation, duodenum-preserving pancreas head resection, ventral pancreatectomy, resection of uncinate process) have been reported [1]. MPP is a relatively new procedure for lesions located in both head and tail of the pancreas. MPP is a combined procedure of pancreaticodudenectomy and distal pancreatectomy, preserving the body of the pancreas to avoid endocrine and exocrine insufficiency. The procedure was first reported by Siassi et al. [2]. It was a metachronous surgery performing pylorus-preserving pancreaticoduodenectomy after previous distal pancreatectomy with splenectomy for pancreatic cancer. In 2007, Miura reported the first case of simultaneous MPP which was performed for ampullary carcinoma in the pancreas head and intraductal papillary mucinous neoplasm (IPMN) in the pancreas tail [3]. Since then, there have only been a few reports of MPP. We herein report a case of MPP for multifocal IPMNs, and describe the surgical procedure.

## A case report

**Fig. 1:**
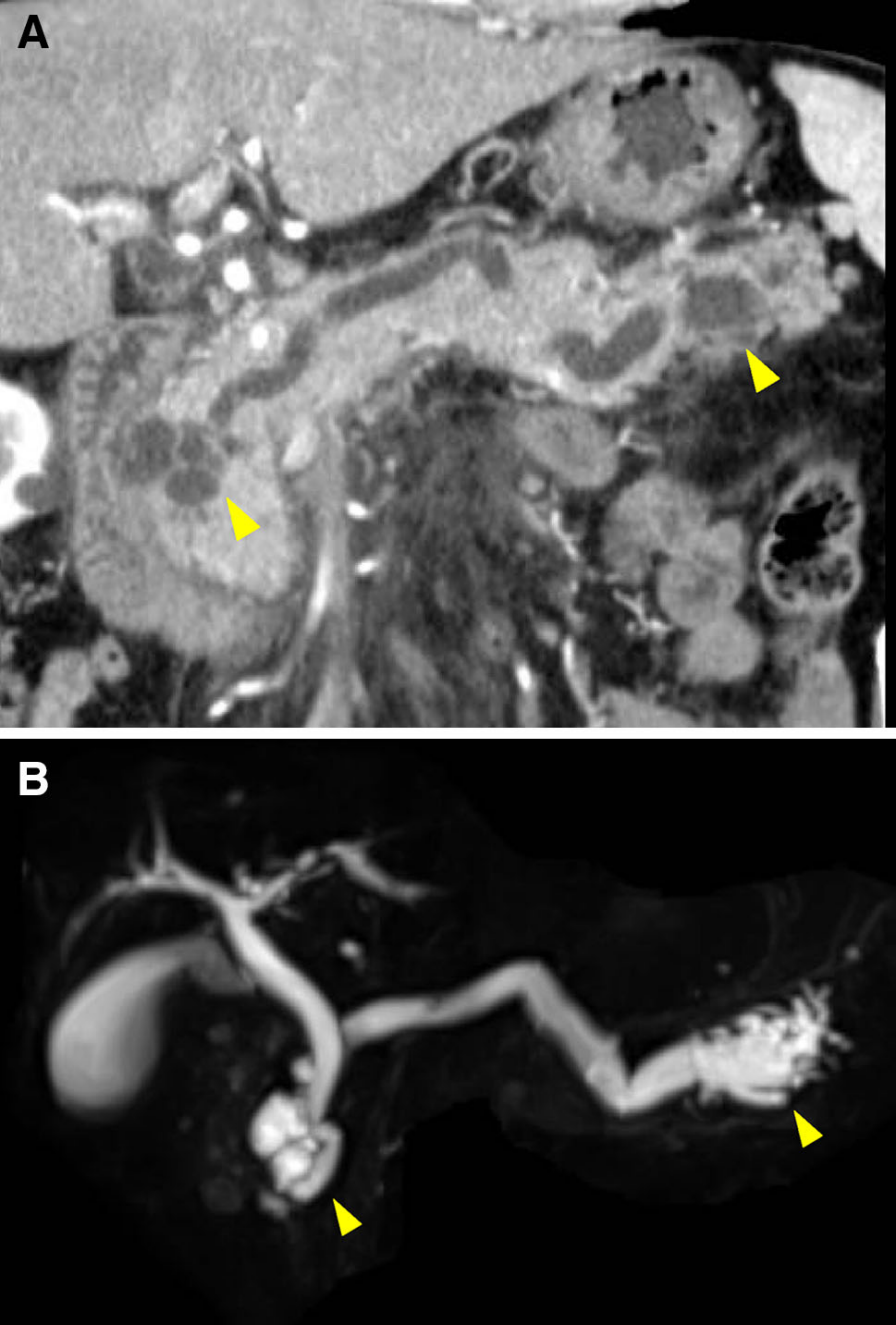
Abdominal CT (**a**) and MRCP (**b**) showed diffuse dilation of the main pancreatic duct, multiple cystic lesions in the pancreatic head, and obvious dilation of the main pancreatic duct in the pancreatic tail

A 76-year-old Japanese man received follow-up treatment for branch duct type IPMNs of the pancreas in the pancreatic head first diagnosed in 2003. In 2011, the known lesion in the pancreatic head was enlarged, and a main duct type IPMN in the tail of the pancreas had newly appeared following acute pancreatitis. The findings on physical examination were unremarkable. Laboratory tests were as follows: leukocyte count 4,540/μl, hemoglobin 13.6 g/dl, albumin 4.0 g/dl, amylase 216 IU/l, total bilirubin 0.5 mg/dl, aspartate aminotransferase 21 U/l, carcinoembryonic antigen (CEA) 1.7 ng/ml, DUPAN-II <25 U/ml, S-pancreas-1 antigen (Span-1) <13.8 U/ml. Computed tomography (CT) and magnetic resonance cholaongiopancreatography (MRCP) showed a 3-cm multicystic mass in the pancreatic head, dilated main pancreatic duct in full length, and a cystic lesion in the pancreatic tail (Fig. 1a, b). Endoscopic retrograde pancreatography showed the wide open papilla of Vater and mucinous outflow from the orifice. Also, cytology of pancreatic juice was class 3. 2-[18F]-fluoro-2-deoxy-d-glucose (FDG) positron emission tomography and CT (PET/CT) did not show abnormal accumulations in the corresponding areas. Preoperative diagnosis was IPMNs of the pancreas with head and tail lesions.

**Fig. 2:**
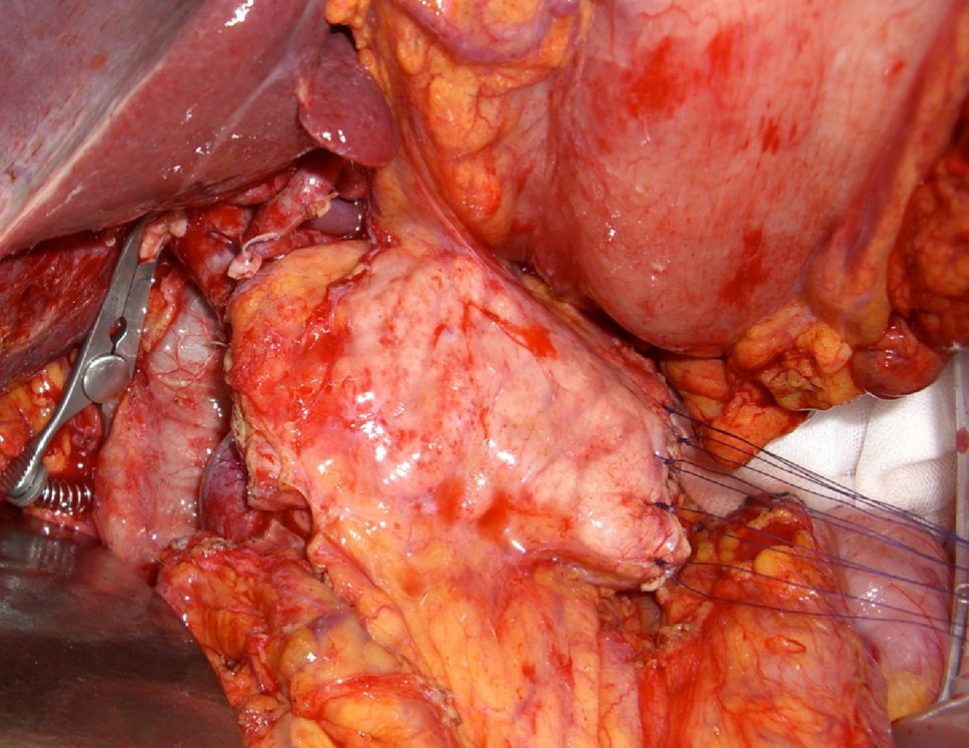
Intraoperative finding. Eight centimeters of pancreatic body was preserved

MPP was performed. An upper abdominal incision was made. The pancreatic head adhered to the surrounding tissue due to previous acute pancreatitis. Distal pancreatectomy with splenectomy was first carried out. The splenic artery was ligated and divided 3 cm distal to the bifurcation of the celiac axis, and the dorsal pancreatic artery was preserved. Pancreatic parenchymal transaction line was confirmed with intraoperative ultrasonography. The distal pancreatic parenchyma was transected at 3 cm proximal to the cystic lesion in the pancreatic tail. Next, subtotal stomach-preserving pancreaticoduodenectomy was carried out. Above the superior mesenteric vein, the proximal pancreatic parenchyma was transected. Intraoperative fresh frozen sections of both pancreatic stumps was negative for pancreatic intraepithelial neoplasia (PanIN). Eight centimeters of the pancreatic body was preserved (Fig. 2). Doppler ultrasonography showed arterial blood supply to the remnant pancreas. Reconstruction involved pancreaticojejunostomy, end-to-side hepaticojejunostomy, and antecolic end-to-side gastrojejunostomy. Distal pancreatic stump was sutured using the fish mouth procedure.

Upon microscopic examination, pathology diagnosis was intraductal papillary mucinous adenoma for the pancreatic head lesion, and non-invasive intraductal papillary mucinous carcinoma for the pancreatic tail lesion. Pancreatic epithelial cells in both pancreatic stumps showed no atypia.

Postoperative course was uneventful, and the patient was discharged 21 days after surgery. The patient maintained good glucose tolerance without insulin administration, and has remained well with no evidence of recurrence at 9-month follow-up.

## Discussion

In this case, we performed MPP, which is a combined procedure of subtotal stomach-preserving pancreaticoduodenectomy for branch duct type IPMN in the pancreatic head and distal pancreatectomy with splenectomy for main duct type IPMN in the pancreatic tail, with 8 cm of the pancreatic body preserved. IPMNs of the pancreas are mucin-producing pancreatic neoplasms, with prominent intraductal growth and frequent papillary architecture. With the advance of radiographic imaging, recently, the diagnosis of IPMNs has been increasing. Of IPMNs found, 39–62 % were multifocal and located in distant segments of the pancreas [4]. International consensus guidelines suggest standard pancreatectomy and lymph node dissection when invasive carcinoma is suspected [5]. Moreover, parenchyma-preserving pancreatectomy is proposed for IPMNs as for other benign or low malignant tumor [5]. However, the oncological and functional outcome of parenchyma-preserving pancreatectomy for IPMNs is uncertain, and surgical indication is still controversial. The appropriate surgical approach remains unclear, especially in patients with multifocal IPMNs located in both the head and tail of the pancreas.

Previous reports of simultaneous MPP are listed in Table 1 [3, 6–11]. Fifteen cases of simultaneous MPP have been reported. The morbidity and the mortality was 53, 0 %, respectively. Pancreatic fistula occurred in six cases (40.0 %). One patient had transient peritoneal bleeding and splenic hematoma after spleen-preserving MPP, which was managed with blood transfusion and angiographic embolization [7]. Another patient had postoperative bleeding at pancreatic stump 2 days after surgery, and had undergone reoperation [8]. Six patients (40 %) had postoperative diabetes; five patients required insulin administration, and one patient was free from insulin. There was no case of brittle diabetes, which is hard to control.

**Table 1:** Previous report of simultaneous middle preserving pancreatectomy

Authors (references)	Histology (head/tail)	Early complication	Postoperative diabetes	Outcome
Miura et al. [3]	AC/IPMN	PF	Yes	6 m alive
Partelli et al. [6]	NET/NET	PF	No	118 m alive
	NET/NET	No	No	22 m alive
	IPMN/IPMN	No	Yes	20 m alive
	IPMN/CP	No	No	18 m alive
	RC/CP	No	Yes	14 m alive
Sperti et al. [7]	IPMN/CP	Bleeding	Yes	11 m alive
Ohzato et al. [8]	RCC/RCC	Bleeding	Yes	30 m alive
Chen et al. [9]	AC/SPT	No	No	6 m alive
Noda et al. [10]	CC/NET	PF	No	1 m alive
Horiguchi et al. [11]	IPMN/ML	PF	No	16 m dead
	NET/NET	PF	No	77 m alive
	IPMN/IPMN	PF	Yes	14 m alive
	BDC/IPMN	No	No	7 m alive
Our case	IPMN/IPMN	No	No	9 m alive

*AC* ampullary carcinoma, *BDC* bile duct cancer, *CC* colon cancer, *CP* chronic pancreatitis, *IPMN* intraductal papillary mucinous neoplasm, *m *months, ML malignant lymphoma, *NET* neuroendocrine tumor, *PF* pancreatic fistula, *RC* retention cyst, *RCC* renal cell carcinoma, *SPT* solid pseudopapillary tumor

We compare MPP with other pancreatic surgeries. Rates of pancreatic fistula, morbidity, and mortality are 9–30, 38–44 and 1–4 % in pancreaticoduodencetomy [12, 13], 0–61, 13–64 and 0–4 % in distal pancreatectomy [14, 15], 8–50, 38–62 and 0–2 % in middle pancreatectomy [16, 17], 0, 15–69 and 2–5 % in TP [18, 19], respectively. MPP has two potential sources of pancreatic fistula, pancreatointestinal anastomosis and distal pancreatic stump. The rate of pancreatic fistula and morbidity of MPP tend to be higher than those of pancreaticoduodenectomy, but similar to those of other procedures. In the context of morbidity and mortality, in our opinion, MPP can be considered an alternative procedure to TP.

In view of remnant pancreatic volume after surgery, it has been suggested that 10–25 % of the pancreatic parenchyma should be preserved to maintain pancreatic function [20]. About 5–6 cm of the pancreatic parenchyma was preserved in previously reported MPP [3, 6–11]. Miura et al. [3] proposed preserving >25 % of the pancreas in MPP. In the current case, we preserved 8 cm of the pancreatic body to avoid endocrine or exocrine insufficiency after surgery.

With regard to the surgical procedure, we need to preserve the dorsal pancreatic artery in MPP. Gastroduodenal artery, inferior pancreaticoduodenal artery, and splenic artery are divided. As a result, blood supply to the preserved pancreatic body depends mainly on the dorsal pancreatic artery originating from the proximal splenic artery or common hepatic artery. There were no reports of postoperative pancreatic infarction after MPP. Decreased blood flow to the remnant pancreas may cause pancreatic fistula or long term pancreatic exocrine and endocrine disturbance. Therefore, care must be taken to preserve the dorsal pancreatic artery. Consequently, lymph node dissection around the celiac axis or splenic artery is limited. Because of the limitations of lymph node dissection and sufficient surgical margin, MPP should not be performed for pancreatic ductal adenocarcinoma (PDAC) in the pancreatic tail. We think benign or low-malignancy lesions, including IPMNs, and metastatic tumors, are an adequate surgical indication for MPP. In addition, we think taking intraoperative frozen sections is essential to prevent tumor relapse. If the frozen section is positive, subsequent additional resection or conversion to TP would be considered.

Regarding oncological outcome, one patient died of malignant lymphoma 16 months after surgery [11]. Nine patients underwent MPP for IPMNs. No recurrence of IPMNs was observed in these nine cases at a median follow-up of 14 months. IPMNs are risk factors for PDAC. After MPP, appropriate follow-up is needed for recurrence of IPMNs or new occurrence of PDAC. It is difficult to define oncological outcomes of MPP due to the small number of patients. A longer follow-up and larger series of patients are needed to confirm results.

In conclusion, we reported a case of MPP for multifocal IPMNs of the pancreas. Thus, surgeons should take MPP into consideration, when the lesions involve the head and tail of the pancreas. MPP is a safe and feasible procedure for benign and low-malignancy tumors including IPMNs of the pancreas.

## Abbreviations

CEA: Carcinoembryonic antigen
CT: Computed tomography
FDG: 2-[18F]-Fluoro-2-deoxy-d-glucose
IPMN: Intraductal papillary mucinous neoplasm
MPP: Middle-preserving pancreatectomy
MRCP: Magnetic resonance cholaongiopancreatography
PDAC: Pancreatic ductal adenocarcinoma
PET/CT: Positron emission tomography and computed tomography
Span-1: S-pancreas-1 antigen
TP: Total pancreatectomy
